# Plant Chemistry Determines Host Preference and Performance of an Invasive Insect

**DOI:** 10.3389/fpls.2020.594663

**Published:** 2020-11-06

**Authors:** Dingli Wang, Lifeng Zhou, Qiyun Wang, Jianqing Ding

**Affiliations:** State Key Laboratory of Crop Stress Adaptation and Improvement, School of Life Sciences, Henan University, Kaifeng, China

**Keywords:** Phthorimaea operculella, oviposition preference, development performance, Solanaceae, plant chemicals

## Abstract

Understanding how host plant chemistry affects invasive insects is crucial for determining the physiological mechanism of host use and predicting invasive insect outbreak and damage on hosts. Here, we examined the effects of plant nutrition and defensive chemicals on host preference and performance of adults and larvae of the invasive potato tuberworm, *Phthorimaea operculella* (Zeller; Lepidoptera: Gelechiidae), on four native (*Solanum tuberosum*, *Nicotiana tabacum*, *Datura stramonium*, and *Solanum lycopersicum*) and three new (*Solanum melongena*, *Physalis alkekengi*, and *Lycium barbarum*) host plants. We found that adults preferred to oviposit on *S. tuberosum* and *N. tabacum* leaves and the soil around these native host plants over other hosts. Larvae performed well on *S. tuberosum* and *N. tabacum*, reaching higher pupa weight and having better survival. Larvae performed poorly on *S. melongena*, *S. lycopersicum*, *P. alkekengi*, *D. stramonium*, and *L. barbarum*, with lower pupa weight and lower survival. *Solanum tuberosum* and *N. tabacum* had higher leaf soluble proteins than other plants and lower leaf total phenolics than *S. lycopersicum*, *D. stramonium*, and *L. barbarum*. Moreover, carbon content and soluble protein were positively associated with larval survival, while defensive traits (lignin and total phenolics) negatively affected larval survival. These findings provide insights into understanding of biochemical mechanisms of interactions between invasive insects and host plants, indicating the importance of considering plant chemistry when assessing invasive insect host use and damage.

## Introduction

Biological invasions threaten global economic development and ecological and food security, especially when invasive pest species cause loss of crop yields (Huber et al., 2002; Pimentel et al., 2005; Worner and Gevrey, 2006). The introduction, establishment, and colonization of alien insect species are determined by many factors, for example, host availability and food quality (Burrack et al., 2013; Kenis et al., 2016; Cowie et al., 2019). In such invasive processes, host plant species can significantly influence adult oviposition preference and larval developmental performance of phytophagous insect (Abbes et al., 2016). Moreover, invasive pest insects are usually distributed in areas with different plant hosts, likely leading to differences in the insect population densities (Nylin et al., 2014). Therefore, estimating the preference and performance of invasive pest insects on various host plants could help predict their population dynamics and damage potential in invaded areas.

Plant primary and secondary metabolisms determine host plant quality and potentially influence feeding performance and life history traits of phytophagous insect. Many studies suggest that high concentrations of defensive chemicals (i.e., alkaloids, glucosinolates, terpenoids, and phenolics) with toxic, antifeedant, or repellent properties can significantly reduce insect performance (Bezemer et al., 2004; Mumm et al., 2008; Yan et al., 2018; Fuchs et al., 2020), while relatively high amounts of nitrogen are beneficial for insect development (Awmack and Leather, 2002). In addition, plant structural components of cell walls, cellulose and lignin, play essential roles in plant defenses as constitutive barriers. For instance, plant cellulose content can effectively predict plant defense traits (i.e., leaf toughness) and is negatively associated with some types of insect feeding, such as leaf miners (Kitajima et al., 2012; Fuentealba et al., 2020). Therefore, understanding of how these phytochemicals affect invasive insect performance is critical in predicting invasive insect invasiveness.

Potato tuberworm, *Phthorimaea operculella* (Zeller; Lepidoptera: Gelechiidae), is an important invasive insect originating in South America. It attacks many solanaceous crops and wild plants such as potato (*S. tuberosum*), tobacco (*N. tabacum*), tomato (*S. lycopersicum*), eggplant (*S. melongena*), datura (*D. stramonium*), wolfberry (*L. barbarum*), and the genera *Physalis* in temperate and subtropical regions (Rondon, 2010). *Solanum tuberosum*, *N. tabacum*, *D. stramonium*, and *S. lycopersicum* are native hosts for this insect, while the others are new hosts of the insect in the invaded areas, i.e., *S. melongena*, *P. alkekengi*, and *L. barbarum* are native to Asia. Adults of potato tuberworm lay eggs in foliage, exposed tubers, or soil surrounding host plants. Larvae mine leaves, petioles, stems, and exposed tubers in the field. With the increase in global warming, the potato tuberworm has invaded more than 90 countries because of its temperature dependence and a high adaptability to diverse conditions (Rondon, 2010; Kroschel and Schaub, 2013). Furthermore, the accelerated invasion of potato tuberworm might also depend on broad host range and is closely related to the larval developmental performance on multiple hosts (Kroschel and Schaub, 2013). Thus, it is important to determine the effects of different host plants on the performance of potato tuberworm, which in turn should improve the integrated management of this pest.

The performance of potato tuberworm larvae may be strongly influenced by host plant chemistry. Pacifico et al. (2019) found that α-chaconine and caffeic acid in potato tuber skin significantly affected its larval survival. Tomato fruit biochemical (α-tomatine) and physical (fruits size and maturity) properties may also impact the growth, development, and survival of *P. operculella* larvae (Mulatu et al., 2006). Study of the effects of four host plants and two nonhost plants on the behavior of newly hatched larvae of *P. operculella* (Varela and Bernays, 1988) showed that leaf surface extracts might affect larval hatch rates from soil-laid eggs and subsequent larval dispersal onto plants. Plant odors may also important in mediating host plant location and ovipositional selection by adult tuber moth (Fenemore, 1988). Although many previous studies have addressed the importance of plant chemistry in determining *P. operculella* performance, it is still not clear how primary metabolism and defensive chemicals of host plants affect its host preference and performance and then promote its invasiveness.

In this study, we ask the following questions: (1) what are the differences in performance and preference of *P. operculella* among native and novel host plants? and (2) does host chemistry lead to a difference in the larval performance of *P. operculella*? To answer these questions, we examined the oviposition preference of adult tuber moth and the larval developmental characteristics on seven solanaceous plants. We then evaluated the relationship between the phytochemistry and insect performance by determining primary and secondary metabolisms in our test plant species.

## Materials and Methods

### Rearing of Potato Tuberworm

We obtained larvae of potato tuberworms from potato fields in Xuanwei City (Yunnan Province, China). They were brought back to the laboratory and reared on potato tubers in wooden cages (40 × 40 × 40 cm) held at 27 ± 2°C, 70–80% RH and a 12:12 h L:D photoperiod. They pupated in sand in the cages. After adults emerged, we collected 50 pairs of adults and placed them for mating and oviposition in plastic containers (height: 25 cm and diameter: 20 cm) with 10% honey water and provided with filter paper as the oviposition substrate (Wang et al., 2020). Eggs were placed in zip-lock bags and hatched in above experimental conditions. Newly hatched larvae (within 12 h) were used in our study.

### Plant Materials

Seeds of eggplant (*S. melongena*) and tomato (*S. lycopersicum*) were obtained from the Chinese Academy of Agricultural Sciences. Seeds of tobacco (*N. tabacum*) were obtained from Henan Agricultural University. Seeds of *P. alkekengi*, datura (*D. stramonium*) and wolfberry (*L. barbarum*) were obtained from Shouguang Seed Center in Shandong, China. Potato seed-tubers were obtained from the Zhangzhou Vegetable Research Institute. All seed plants were planted in growing medium (half field soil and half sphagnum peat moss) for germination and initial growth in an open-sided greenhouse with natural temperature and light. After 2 weeks of growth, similarly sized seedlings were selected from each species to transplant individually into pots (height: 18 cm and diameter: 24 cm) filled with a mixture of half field soil and half sphagnum peat moss. For potato plants, tubers were cut into about 25 g pieces, each with an eye bud, and then planted 8 cm deep into pots of the same size and soil as above. All plants were covered with fabric (nylon netting) in a greenhouse (10 × 5 × 2 m) at Henan University, Kaifeng, China (34°49'N, 114°18'E). Plants were not fertilized but watered as necessary. Plants that had been growing for 40 days were used throughout the experiment.

### Oviposition Preference Experiment

To test the ovipositional preference of *P. operculella* adults, the seven selected solanaceous plants were individually evaluated in no-choice tests. Experiments were performed in nylon cages (20 cm diameter and 80 cm height) containing one single plant. We exposed two pairs of adult moths to each host plant, allowing them to mate and lay eggs freely, and provided them with 10% honey solution. Four days later, we counted the number of eggs laid in plant leaves and nylon cage using a magnifier. For the number of eggs in the soil around the plant, we first collected 1-cm-deep potted soil and put them in Petri dishes (diameter: 15 cm). We then determined the number of eggs under a light microscope using the methodology suggested by Karlsson (2009). Each cage was considered as one replicate, and there were 10 replicates per host plant.

### Larval Development Experiment

To measure the developmental performance of *P. operculella* larvae on plants, we placed five newly hatched larvae on the upper surface of five leaves of each solanaceous plant using a soft brush, until larvae were situated on the leaves. Each leaf was covered with a small nylon bag (15 cm long and 10 cm wide) to prevent the larvae from moving or falling. In this experiment, each plant was used as one replicate, with 10 replicates per plant species. One week after infestation of plants, to prevent the larvae from pupating in the soil, each potted plant was covered with a plastic bag that was sealed at the plant stem. Then each pot was placed inside a larger plastic pot (50 cm diameter and 18 cm height) with 2 cm of sterile sand, which was intended as the larval pupation site. Ten days after initial infestation, we removed bags and examined pupae from the sterile sand daily. All pupae recovered were weighed and placed individually in Petri dishes (5 cm diameter and 1 cm height), lined with tissue paper, and held for adult emergence, which were then sexed. Accordingly, we determined the developmental time of each insect in its larval and pupal stages.

### Plant Chemistry

Ten randomly selected samples from each plant species were collected and analyzed for the levels of primary and secondary metabolites. Leaves of each healthy plant were cut off and carefully cleaned with water. Then all leaves were dried at 45°C for 5 days and ground to powder. The samples were then analyzed for total carbon content (C) and total nitrogen content (N) using an elemental auto-analyzer (Vario MAX CN; Elementar, Hanau, Germany). According to Elleuch et al. (2007), the soluble sugar content was determined by spectrophotometry (Thermo Scientific GENESYS 10S, Waltham, MA, USA) at 630 nm wavelength. Plant soluble protein levels were determined following Bradford (1976). Plant cellulose and lignin levels were analyzed by spectrophotometry at 620 and 280 nm wavelengths, following Updegraff (1969) and Morrison (1972). Analysis of total phenolics was based on the method of Ainsworth and Gillespie (2007), using gallic acid as the standard and absorbance determined by spectrophotometer at 765 nm.

### Data Analysis

Differences in the number of eggs laid, larval survival rate, C and N contents, C:N ratio, levels of soluble sugar, soluble protein, cellulose lignin, and total phenolics among the seven solanaceous plants were analyzed with one-way ANOVA, with *post-hoc* Tukey’s HSD test for multiple comparisons. To assess differences in insect response to host plants and insect sex, we used two-way ANOVAs to analyze larval and pupal developmental time and pupal weight, with host plants and insect sex as the main factors. Then, we used one-way ANOVA, with *post-hoc* Tukey’s HSD test to evaluate the effects of host plants on female and male performance, respectively. An independent-sample *t*-test was performed to test for differences in pupal weight between females and males on each host. Moreover, to estimate the role of plant chemicals in the effects of host plants on the larval survival in the ANOVAs, we performed separate regression analyses to determine the relationships between C content, N content, C:N ratio, levels of soluble sugar, soluble protein, cellulose, lignin, and total phenolics, with larval survival rate. In all analyses, data were checked and satisfied the assumption of normality and homogeneity of variances. We performed all data analyses with software R, version 3.4.2 (R Development Core Team, 2017).

## Results

### Ovipositional Preference and Development Performance

**Figure 1:**
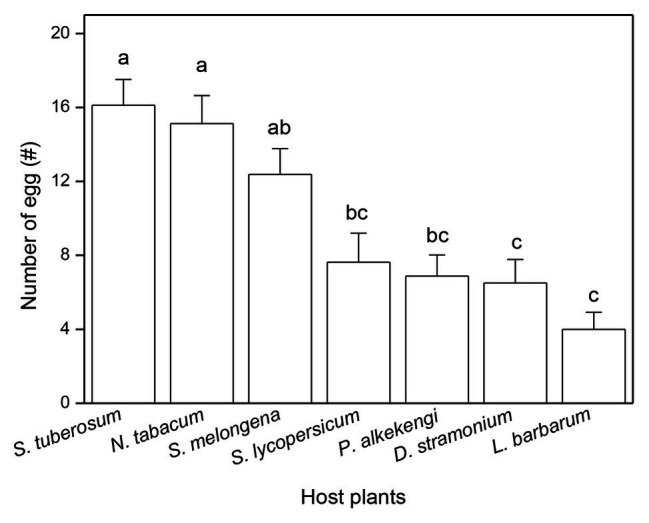
Total number of eggs laid by *Phthorimaea operculella* adults on leaves and soil around plant of seven solanaceous plants. Data are means ± SE. Different letters show significant differences in different host plants (*p* < 0.05) based on *post hoc* Tukey’s HSD test.

The number of eggs laid (on leaves, soil, or the cage netting) was significantly affected by the host plant species in no-choice experiments (Table 1). The highest number of eggs was laid on leaves of *N. tabacum* and the lowest number of eggs was laid on leaves of *L. barbarum* (Table 1). The number of eggs in the soil around *S. tuberosum* was the largest and the number of eggs in the soil around *L. barbarum* was the lowest (Table 1). The greatest number of eggs that were laid on the nylon netting occurred with *L. barbarum* and the fewest with *N. tabacum* (Table 1). However, there was no significant difference in the total number of eggs of three ovipositional sites among seven tested plants (Table 1). Moreover, ovipositional preference, as determined by the total number of eggs laid on the plant leaf and the soil around the plant, was also significantly influenced by plant species (F_6,49_ = 12.424, *p* < 0.0001; Figure 1). The adult tuber moths were most likely to lay eggs on *S. tuberosum* and least likely to do so on *L. barbarum* (Figure 1).

**Table 1:** One-way ANOVA analyses for no. of eggs of *Phthorimaea operculella* adult in different ovipositional sites (leaf, soil, and net) for seven solanaceous plants.

Species	No. of eggs (leaf)	No. of eggs (soil)	No. of eggs (net)	No. of eggs (total)
*Solanum tuberosum*	2.375 ± 0.565	13.750 ± 1.175	18.250 ± 1.544	34.375 ± 2.521
*Nicotiana tabacum*	3.250 ± 0.921	11.875 ± 1.060	16.000 ± 1.927	31.125 ± 1.959
*Solanum melongena*	2.000 ± 0.756	10.375 ± 1.084	17.250 ± 2.366	29.500 ± 2.375
*Solanum lycopersicum*	0.875 ± 0.398	6.750 ± 1.509	21.875 ± 1.575	29.625 ± 3.218
*Physalis alkekengi*	0.875 ± 0.515	6.000 ± 1.000	23.250 ± 2.821	30.125 ± 2.662
*Datura stramonium*	0.500 ± 0.378	6.000 ± 1.000	23.000 ± 2.315	29.500 ± 3.240
*Lycium barbarum*	0.375 ± 0.263	3.625 ± 0.800	25.625 ± 1.267	29.625 ± 1.762
df	6,49	6,49	6,49	6,49
F	3.496	11.071	3.104	0.474
*p*	**0.006**	**<0.0001**	**0.012**	0.824

All data are mean ± SE. Significant results are in bold.

**Figure 2:**
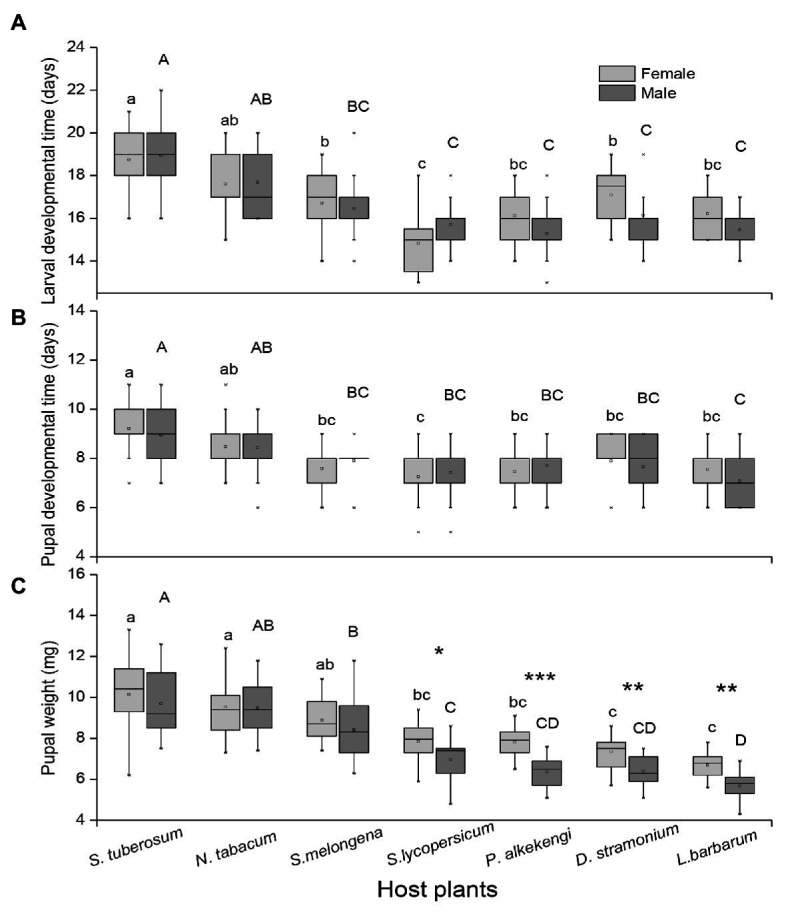
Differences in **(A)** larval developmental time, **(B)** pupal developmental time, and **(C)** pupal weights of female and male *Phthorimaea operculella* among seven solanaceous plants. Gray bars indicate females and black bars indicate males. Data are means ± SE. Different letters show significant differences in different host plants (*p* < 0.05) based on *post hoc* Tukey’s HSD test, small letters indicate difference in female development performance among host plants, and big letters indicate difference in male development performance among host plants. Asterisks indicate difference in pupa weight between female and male with same host plant. *p*: ^*^ ≤ 0.05, ^**^ ≤ 0.01, and ^***^ ≤ 0.001.

There was a significant effect of host plant species on larval developmental time, pupal developmental time, and pupal weight, and only pupal weight was significantly affected by insect sex. However, there was no significant influence of interactions of host plants and sex on insect performance (Table 2; Figure 2). The longest and shortest larval developmental time were observed on *S. tuberosum* and *S. lycopersicum*, respectively (Figure 2A). The longest pupal developmental time was observed on *S. tuberosum*, followed by *N. tabacum*, while shorter pupal developmental time was observed on *S. melongena*, *S. lycopersicum*, *P. alkekengi*, *D. stramonium*, and *L. barbarum* (Figure 2B). Pupal weight was highest on *S. tuberosum* and lowest on *L. barbarum*. Moreover, there was a significant difference in pupal weight between female and male potato tuberworm in *S. lycopersicum* (*t* = 2.305, df = 31, *p* = 0.028; Figure 2C), *P. alkekengi* (*t* = 5.382, df = 32, *p* < 0.0001; Figure 2C), *D. stramonium* (*t* = 3.224, df = 29, *p* = 0.003; Figure 2C), and *L. barbarum* (*t* = 3.298, df = 20, *p* = 0.004; Figure 2C).

**Table 2:** Two-way ANOVAs for larval and pupal developmental time and pupal weight of *Phthorimaea operculella* of different sexes in leaf tissues with different host plants.

Source of variation		Larval developmental time		Pupal weight		Pupal developmental time	
	df	F	*p*	F	*p*	F	*p*
Host plants (HP)	6	30.114	**<0.0001**	46.991	**<0.0001**	14.001	**<0.0001**
Sex (S)	1	1.54	0.216	22.009	**<0.0001**	0.091	0.764
HP × S	6	1.814	0.097	1.333	0.243	0.611	0.721

Significant results are in bold.

**Figure 3:**
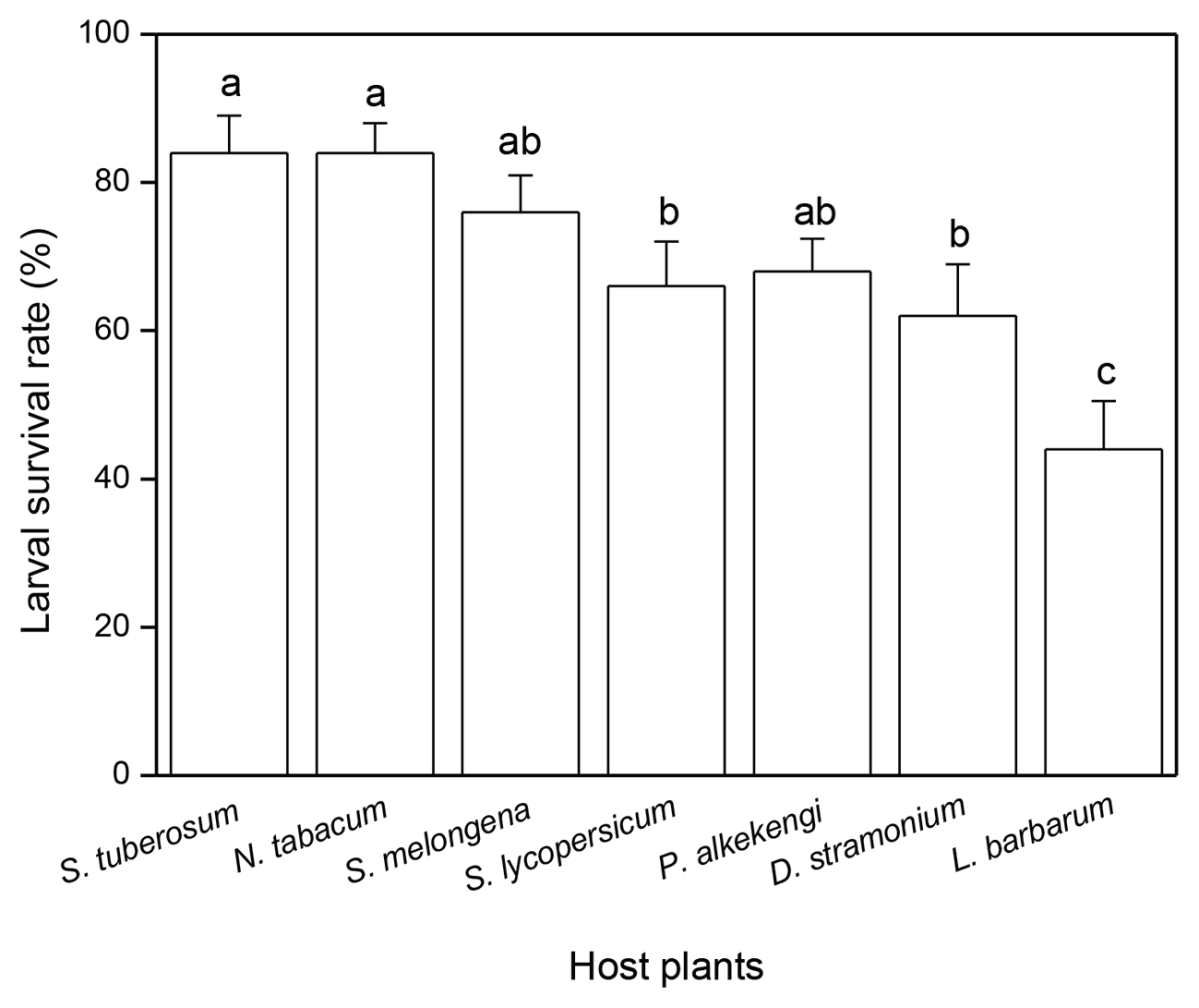
Effects of host plant species on larval survival rate of *Phthorimaea operculella*. Data are means ± SE. Different letters show significant differences in different host plants (*p* < 0.05) based on *post hoc* Tukey’s HSD test.

Larval survival rate was significantly influenced by host plant (F_6,63_ = 6.496, *p* < 0.0001, Figure 3). The highest larval survival rate was observed in *S. tuberosum* (84%) and *N. tabacum* (84%), followed by *S. melongena* (76%), *S. lycopersicum* (66%), *P. alkekengi* (68%), and *D. stramonium* (62%), while the lowest larval survival rate was observed in *L. barbarum* (44%) (Figure 3).

### Plant Nutrients and Defensive Chemicals

**Figure 4:**
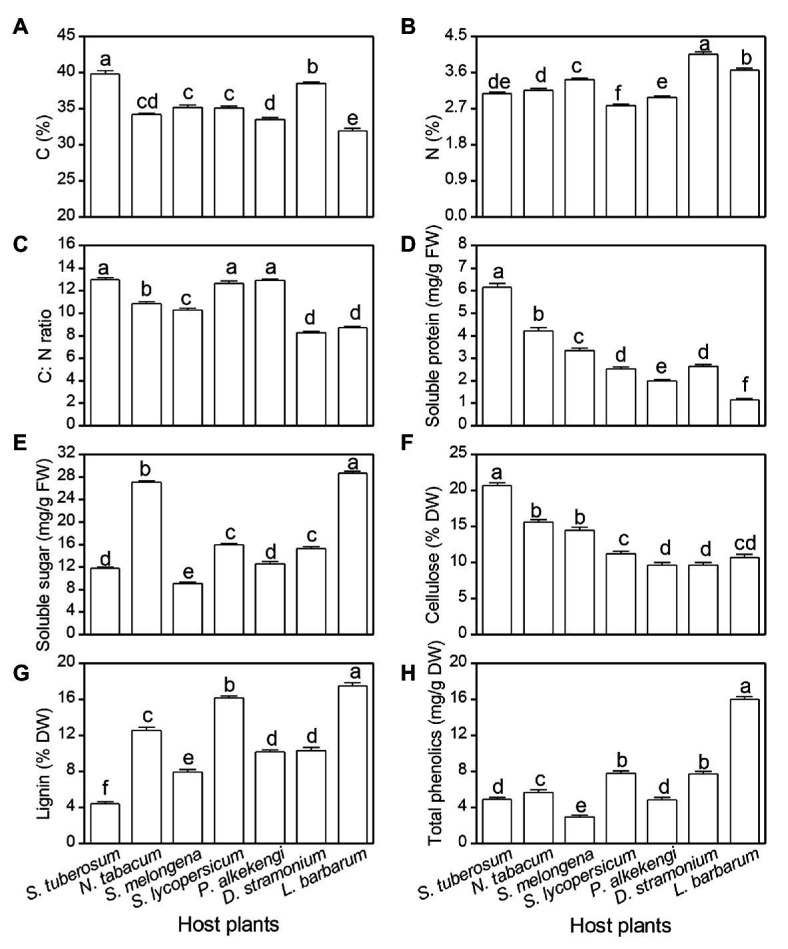
Difference in plant chemicals among seven solanaceous plants. Carbon content **(A)**, nitrogen content **(B)**, C:N **(C)**, soluble protein **(D)**, soluble sugar **(E)**, cellulose **(F)**, lignin **(G)**, and total phenolics **(H)**. Data are means ± SE. Different letters show significant differences in different host plants (*p* < 0.05) based on *post hoc* Tukey’s HSD test.

All plant chemicals were significantly different among the seven tested host plants (Table 3; Figure 4). The highest and lowest plant C content was observed on *S. tuberosum* (39.8%) and *L. barbarum* (31.9%), respectively (Figure 4A). The highest N content was observed on *D. stramonium* (4.1%), and the lowest content was observed on *S. lycopersicum* (2.8%) (Figure 4B). The highest C:N ratio was observed on *S. tuberosum*, *S. lycopersicum*, and *P. alkekengi* (12.9, 12.6, and 12.8, respectively), followed by *N. tabacum* (10.9) and *S. melongena* (10.3), and the lowest was observed on *D. stramonium* (8.3) and *L. barbarum* (8.7; Figure 4C). The soluble protein of *S. tuberosum* was highest, while it was lowest on *L. barbarum* (Figure 4D). However, the soluble sugar of *L. barbarum* was highest, while it was lowest on *S. melongena* (Figure 4E). The cellulose level was highest on *S. tuberosum* and lowest on *P. peruviana* and *D. stramonium* (Figure 4F). The highest lignin and total phenolics was observed on *L. barbarum*, and the lowest lignin was observed on *S. tuberosum* and lowest total phenolics was observed on *S. melongena* (Figures 4G,H).

**Table 3:** Results of one-way ANOVAs for plant chemicals of seven tested plants.

Response variable	F	df	*p*
C content (%)	77.517	6, 63	**<0.0001**
N content (%)	139.294	6, 63	**<0.0001**
C:N ratio	142.129	6, 63	**<0.0001**
Soluble protein (mg/g FW)	193.727	6, 63	**<0.0001**
Soluble sugar (mg/g FW)	515.038	6, 63	**<0.0001**
Cellulose (%)	122.591	6, 63	**<0.0001**
Lignin (%)	248.709	6, 63	**<0.0001**
Total phenolics (mg/g DW)	290.238	6, 63	**<0.0001**

FW indicates fresh weight and DW indicates dry weight. Significant results are in bold.

### Relationship of Phytochemistry and Larval Survival

**Figure 5:**
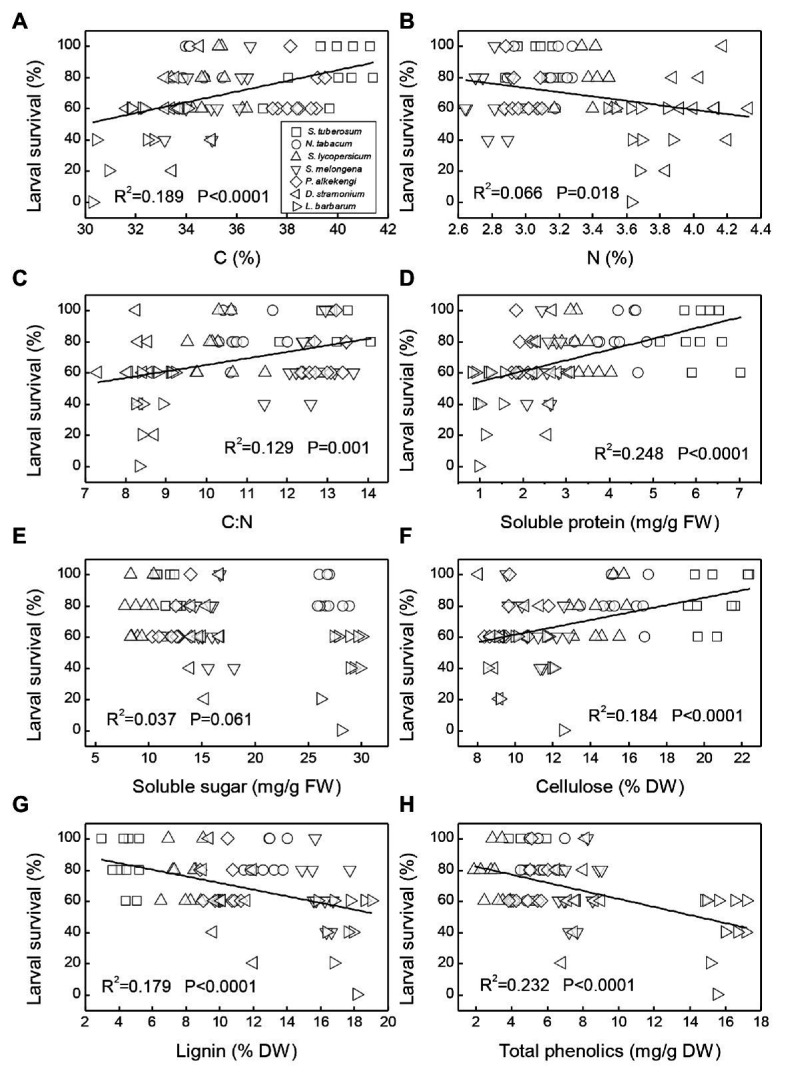
Relationship between larval survival rate of *Phthorimaea operculella* and plant chemicals. Linear regression of larval survival rate on carbon content **(A)**, nitrogen content **(B)**, C:N ratio **(C)**, soluble protein **(D)**, soluble sugar **(E)**, cellulose **(F)**, lignin **(G),** and total phenolics **(H)** with different host plants (□: *Solanum tuberosum*; ○: *Nicotiana tabacum*; △: *Solanum lycopersicum*; ▽: *Solanum melongena*; ◇: *Physalis alkekengi*; ◁: *Datura stramonium*; and ▷: *Lycium barbarum*).

Regressions indicated that seven plant chemicals (C content, N content, C:N, soluble protein, cellulose, lignin, and total phenolics) were significantly associated with larval survival (Figure 5). N content, lignin, and total phenolics had a significant negative relationship with larval survival (Figures 5B,G,H). In contrast, larval survival was positively affected by the content of C, C:N, contents of soluble protein, and cellulose (Figures 5A,C,D,F). However, there was no significant relationship between soluble sugar and larval survival (Figure 5E).

## Discussion

In this study, we examined host use by potato tuberworm from the perspective of plant nutrition and defensive chemicals, by describing the oviposition preference and development performance of this pest on seven solanaceous plants. We found that the number of eggs and larval survival were higher on potato and tobacco plants, which had higher soluble protein and lower total phenolics, while the reverse was true for tomato, datura, and wolfberry. Our results show a plant chemical-dependent host use pattern for this pest species, which will help us to predict the population dynamics of potato tuberworm on these and future new host plants in its invaded ranges.

We found that host plant species significantly affected potato tuberworm oviposition, with the most eggs laid on potatoes and the least on wolfberry. These differences may be due to plant odor or physical and chemical characteristics. Previous studies have suggested that female potato tuber moths prefer to lay their eggs on a rough substrate rather than smooth substrate (Meisner et al., 1974; Traynier, 1975). Oviposition experiments in different plant life stages suggest that the female tuber moth, *Tecia solanivora* (Povolný), tend to lay eggs near mature flowering plants, which may be due to levels of sesquiterpenes compounds, which influence host-finding and oviposition behavior (Karlsson et al., 2009). Fenemore (1979) found that odor of the potato tuber can cause female *P. operculella* to produce more eggs. These plant volatiles may be important signaling factors of host plant quality, likely determining the ovipositional capacity of females in various host plants. In addition, compounds found in plant extracts can also influence oviposition host selection, with female *P. operculella* tending to lay significantly more eggs on substrates with potato plant extract than on other host plant extracts (Traynier, 1975).

We found *P. operculella* pupal weight, larval, and pupal developmental time were significantly affected by the solanaceous host species. In general, larval and pupal development time are the most important parameters to assess a host plant’s suitability to a phytophagous insect (Awmack and Leather, 2002; Saeed et al., 2010; Horgan et al., 2012). Here, larval and pupal developmental time were longest on potato and shortest on tomato and wolfberry. Pupal weight was greatest on potato and least on wolfberry, and larval survival rate was highest on potato and lowest on wolfberry. Previous studies have generally suggested that insects have a high survival rate and faster development time on their preferred host plants (Caparros Megido et al., 2013; Sylla et al., 2019). Inconsistently, our data showed high survival rate but long, not short, larval developmental time for *P. operculella* on their primary hosts, potato and tobacco. Moreover, there were also significant differences in the pupal weight between males and females on tomato, *P. alkekengi*, datura, and wolfberry. These results suggest that plant quality of these unfavorable hosts negatively affects males more than females, especially for pupal weight of the potato tuberworm.

Many studies have suggested that primary chemical compounds involved in plant physiological processes potentially alter developmental performance, reproductive capacity, and morphological characteristics of phytophagous insects (Berenbaum, 1995; Awmack and Leather, 2002; Saeed et al., 2010). In our study, the high pupal weight and survival rate in *S. tuberosum* and *N. tabacum* could be due to high plant nutrition resources (i.e., C content and soluble protein) available to potato tuberworm in their host plants. Indeed, several studies have reported that plant carbon-based nutrients (i.e., sterols and carbohydrates) can positively influence insect performance (Ishaaya et al., 1971; Awmack and Leather, 2002).

Plant anti-herbivore secondary metabolites (i.e., phenolics) and plant structural components (i.e., cellulose and lignin) are important in affecting insect development. For example, lignin, as an important predictor of leaf toughness, can decrease arthropod damage, even at relatively low amounts (Ohmart and Edwards, 1991; Steinbauer et al., 1998; Armani et al., 2020). Our results indicated that lignin content is a significant defense strategy, suggesting that potato tuberworm larvae develop better in *S. tuberosum* plants with low lignin, but worse in *L. barbarum* with high lignin. Indeed, our regression results also showed that lignin content significantly and negatively affected larval survival. Thus, our findings of higher lignin content in *L. barbarum* may explain the lower larval survival and pupal weights on that plant compared with *S. tuberosum*. However, we notice that the R-squared values of the linear regression between lignin and many other chemicals and larval survival were small (Figure 5). It is well-known that phytochemistry not only determines insect performance by affecting insect palatability, but also regulates plant physiology (photosynthesis) and participates in defense against abiotic stresses (salinity, drought, and UV; Evans, 1989; Sharma et al., 2019). Thus, in our study, variation in these chemicals between plant species could only partly be associated with variation in the larval survival.

Phenolic compounds exist in many plants and can act as defensive metabolites involved in plant resistance against insect herbivory (Cheynier et al., 2013; Hinman et al., 2019). In this study, we found a significant and negative correlation between total phenolics and larval survival, suggesting relatively high level of total phenolics may lead to low larval survival in the alternate hosts (*L. barbarum*, *D. stramonium*, and *S. lycopersicum*). However, we only used total phenolics as a plant defense index, various alkaloids from solanaceous plants may also affect the performance of insects. For example, several studies have reported that α-tomatine of *S. lycopersicum* and glycoalkaloids (α-solanine and α-chaconine) of *S. tuberosum* can significantly affect the growth and development performance of insects (Mulatu et al., 2006; Kumar et al., 2016).

Our results have important implications for understanding the phytochemical and physiological mechanisms of potato and tobacco as the best hosts of potato tuberworm and predicting the population dynamics of invasive pest species. Differences in ovipositional preference and the development performance of potato tuberworm depend significantly on plant nutrition and defensive chemicals among the seven tested host plants. This study can help us to better understand the effect of potato tuberworm on Solanaceae crops in invaded areas. We predict potato tuber moth may only outbreak in potato and tobacco growing areas, attacking potato and tobacco. Such effects can be due to high plant nutrition and low defensive chemicals in these two primary hosts. In areas, where there are only its new but minor hosts, e.g., *S. melongena*, *P. alkekengi*, and *L. barbarum*, the insect will perform poorly due to low plant nutrition and high defensive chemicals in these hosts.

## Conclusion

We clearly showed profound differences in adult oviposition and larval developmental performance of the invasive potato tuberworm among the tested solanaceous plants. Such differences depend on host plant quality, demonstrating that plant nutrition (i.e., C content and soluble protein) positively affects larval development and survival. Compared with potato and tobacco plants, new hosts in the introduced range can produce more hard-to-digest compounds (i.e., lignin and total phenolics), which reduce leaf palatability to potato tuberworm. These findings may also improve our understanding of the biochemical mechanisms of the host use of other invasive pest insects.

## Data Availability Statement

The raw data supporting the conclusions of this article will be made available by the authors, without undue reservation.

## Author Contributions

DW and JD designed the experiment and analyzed the data. DW, QW, and LZ conducted the experiment. All authors contributed to the article and approved the submitted version.

### Conflict of Interest

The authors declare that the research was conducted in the absence of any commercial or financial relationships that could be construed as a potential conflict of interest.
